# Occurrence and Correlates of Vitamin D and Iron Deficiency in Children with Autism Spectrum Disorder

**DOI:** 10.3390/nu17172738

**Published:** 2025-08-23

**Authors:** Magdalena Yvonne Koh, Audrey J. W. Lee, Hung Chew Wong, Ramkumar Aishworiya

**Affiliations:** 1 Child Development Unit, Khoo Teck Puat-National University Children’s Medical Institute, National University Hospital, Singapore 119228, Singapore; paearam@nus.edu.sg; 2 Department of Paediatrics, Khoo Teck Puat-National University Children’s Medical Institute, National University Hospital, Singapore 119228, Singapore; audrey_lee@nuhs.edu.sg; 3 Research Support Unit, Yong Loo Lin School of Medicine, National University of Singapore, Singapore 117597, Singapore; medwhc@nus.edu.sg; 4 Department of Paediatrics, Yong Loo Lin School of Medicine, National University of Singapore, Singapore 117597, Singapore

**Keywords:** autism spectrum disorder, iron deficiency, iron deficiency anaemia, picky eating, vitamin D deficiency, vitamin D insufficiency

## Abstract

**Background/Objectives**: This study aimed to determine the occurrence of vitamin D and iron deficiency in children with autism spectrum disorder (ASD) in Singapore and identify correlates of the presence of these deficiencies, if any. **Methods**: This is an observational, cross-sectional, retrospective review of children with a diagnosis of autism, aged 1 to 10 years old, seen at a tertiary developmental paediatric centre from January 2018 to December 2022, with blood investigations completed. Autism diagnosis was determined either clinically by a developmental paediatrician using DSM-5 criteria or using the Autism Diagnostic Observation Schedule (ADOS-2). Children with genetic disorders and chronic medical conditions were excluded. Logistic regression was used to evaluate associations with the deficiencies, and the Bonferroni method was applied on post hoc comparisons. **Results**: The overall sample comprised 241 children (79% males, mean age 4.2 years [SD 2.25]. There were 222 and 236 children who had blood investigations for vitamin D and iron levels performed, respectively. Out of the 222 children whose vitamin D tests were performed, 36.5% had vitamin D insufficiency/deficiency. Iron deficiency occurred in 37.7% for children who had their iron levels tested. There were 122 observations for both iron levels and complete blood count. Out of these, 19 (15.6%) had iron deficiency anaemia. There were no significant correlates for iron deficiency, with picky eating included. **Conclusions**: Vitamin D and iron deficiencies were common in this sample. Clinicians should consider testing for vitamin D and iron for children with ASD, especially for vitamin D in children of Indian ethnicity and older age.

## 1. Introduction

Autism spectrum disorder (ASD) is a developmental condition that involves difficulties in social communication and interaction, restricted interests, and repetitive behaviours [1]. In addition to the core symptoms of autism, several co-occurring conditions are common, including medical conditions involving growth and nutrition [2].

In children with ASD, the common presence of sensory seeking behaviours and rigidity predisposes the child to food selectivity and restricted diet [3], which increases the possibility of nutritional deficiencies [4]. Several clinical practice guidelines for autism emphasise the need to identify and treat nutritional deficiencies as part of holistic care for children with ASD [2]. Among the various nutrients, vitamin D and iron are the most well established in terms of regular testing due to the common occurrence and importance of correction of their respective deficiencies.

Vitamin D is an important nutrient in our body. The well-known function of vitamin D is to maintain an intricate balance of serum calcium and phosphate levels in the body [5]. Apart from roles in motor development, research has also suggested important roles for vitamin D in the immune system as an anti-inflammatory agent and in neurological functioning, including cognition and language functioning in children [6,7,8,9].

There is consistent evidence to suggest higher incidence of vitamin D deficiency in autism as compared to typically developing children, including a recent meta-analysis [10]. Studies, including one in our neighbouring country of Malaysia, have also illustrated occurrence of vitamin D insufficiency/deficiency in a significant proportion of children with ASD ranging from 30 to 57% [11,12]. It is postulated that this is due to food selectivity as well as less time spent partaking in outdoor activities (lesser sunlight exposure for cutaneous vitamin D synthesis) in patients with autism as compared to healthy controls [10]. Studies have also suggested that vitamin D deficiency is correlated with the severity of autism, in terms of autism-specific behaviours [12,13]. There is some evidence to suggest an improvement in such behaviours with vitamin D supplementation, highlighting specific benefits of identifying and correcting a deficiency, if any [12,14]. Despite this, knowledge of vitamin D deficiency among children with ASD in Singapore is not well defined.

Iron is another important mineral, vital for cognitive development in early childhood. Studies have shown that iron deficiency with anaemia is associated with cognitive and behavioural deficits [15]. Iron deficiency is postulated to impair the processes of neurotransmitter metabolism in the brain and thus can have negative consequences on learning, attention, memory, and psychomotor functions [16,17,18]. Previous studies have demonstrated high prevalence of iron deficiency in children with ASD although the rates appear to vary across populations [19,20,21,22]. Prevalence rates vary from 6.6% to 32.0% for iron deficiency with lower but still substantial rates of 4.1% to 15.5% for iron deficiency anaemia among children with ASD in studies from Turkey, Australia, and the United States [19,20,21,22]. These deficiencies are likely related to the food selectivity common in autism, related to strong sensory preferences. To date, little is known about iron status in children with ASD in Singapore. In view of the potential role of iron in neurodevelopment, it is important to know the occurrence of iron deficiency and further evaluate treatment effectiveness in children with ASD to promote better neurodevelopmental outcomes.

This study aims to evaluate the presence of vitamin D and iron deficiency in a sample of children diagnosed with ASD in Singapore, and identify correlates of the presence of these deficiencies, if any. We hypothesised that a substantial proportion (≥20%) of our study sample will have either vitamin D deficiency or iron deficiency and that this will be associated with the presence of restrictive feeding habits. Better understanding and awareness of the occurrence of such deficiencies, and factors that increase their likelihood, can improve clinical care for these children.

## 2. Materials and Methods

### 2.1. Sample

This is an observational, cross-sectional study based on a retrospective review of medical records of children diagnosed with autism aged 1 to 10 years old. These patients were seen at a tertiary developmental behavioural paediatric clinic in Singapore from January 2018 to December 2022. Inclusion criteria included children within the above-mentioned age group who were diagnosed with autism based on evaluation by a developmental paediatrician using DSM-5 criteria or using the Autism Diagnostic Observation Schedule (ADOS-2) [23] and had blood investigations for either iron or vitamin D conducted. Children with chronic medical conditions such as liver, renal, cardiac, or gastrointestinal diseases that could affect absorption and metabolization of vitamin D and iron were excluded. Children with genetic disorders were also excluded. Basic information including demographics was also collected for those children who were seen at the clinic but did not have blood investigations performed, but otherwise met the other inclusion criteria. This was to allow the calculation of the proportion of children who had blood investigations performed. For this study, consent was waived, and the study protocol was approved by the hospital ethical committee.

Data extracted included demographic information, known feeding issues, the results of blood investigations for the presence of vitamin D and iron deficiency, and information on treatment prescribed (if any). Abstraction of data was performed by an independent third party who assisted in identifying patients who met inclusion criteria and retrieving data. In our clinic’s standard clinical workflow, blood investigations for presence of vitamin D and iron deficiency are supposed to be routinely offered for all patients with autism, although this is not mandated, and uptake is based on individual patient/parent preferences. If blood tests are performed, this typically includes a complete blood count, iron panel and vitamin D levels, all performed in the same setting of blood draw.

### 2.2. Criteria for Vitamin D and Iron Deficiency

We used ferritin cutoffs of <12 µg/L for children 0 to 4 year 11 months and <15 µg/L for children ≥ 5 years to define presence of iron deficiency, in accordance with World Health Organization recommendations [24]. As serum ferritin is an acute-phase reactant and may be falsely elevated in children with concurrent inflammation, transferrin saturation was also used to determine iron deficiency. We used transferrin saturations of <15% as a cutoff for iron deficiency [25]. Anaemia is defined as Hb < 11.0 g/dL for preschool children < 5 years old and <12.0 g/dL for children 5 to 11 years old, in accordance with recommended criteria [21].

Vitamin D status was based on our hospital laboratory cut-off values; deficiency is defined by 25-hydroxyvitamin D [25(OH)D] < 10 µg/L and insufficiency as 25(OH)D between 10.1 and 29.9 µg/L. Blood investigations were carried out at our hospital biochemistry laboratory using standard measurements.

### 2.3. Factors Associated with Deficiencies

We examined picky eating, race, gender, and age as potential correlates for the presence of these deficiencies. Picky eating was defined as having specific preferences in diet, beyond that expected for age, established following clinical evaluation and history-taking by the developmental behavioural paediatrician, and documented in the medical records of the patient.

### 2.4. Statistical Analysis

Data was analysed using IBM SPSS version 29. Logistic regression was used to evaluate the associations of the demographics and other characteristics with insufficiency/deficiency of vitamin D, iron deficiency, and iron deficiency anaemia. For the unadjusted analyses involving the association of race with insufficiency/deficiency of vitamin D, iron deficiency, and iron deficiency anaemia, the Bonferroni method was applied to the post hoc comparisons. Statistical significance level was set at 5%.

## 3. Results

### 3.1. Characteristics of the Study Population

The final study sample comprised 241 children. This was derived from a total of 1014 children with ASD who were seen at the unit within that time, of which 241 fulfilled the inclusion criteria. Among the 1014 records, we noted that 278 (27.4%) were offered blood tests for nutritional deficiencies by their physician. The study sample comprised 241 children, 79% being male, with a mean age of 4.2 years old (SD 2.25) and interquartile range of 2.4 to 6 years old. The demographic characteristics of the sample can be seen in Table 1. The ethnic distribution in the sample mirrors that of the multi-ethnic population of Singapore. The gender distribution is in keeping with that for autism incidence in general.

Comparing the characteristics between those who have had blood investigations performed versus those who did not, we noted that 48.9% (N = 89) of those who had blood investigations were picky eaters compared to 27.8% (N = 138) being picky eaters of those who did not have investigations. The rest of the demographic characteristics between the two groups were similar (refer to Supplementary Table S1).

There were 222 and 236 children who had blood investigations for vitamin D and iron levels performed, respectively; while the majority had both investigations performed, 5 children who did not have vitamin D testing had investigations performed for iron levels. Among those who had investigations performed for iron deficiency, there were 122 observations for both iron levels and complete blood count.

### 3.2. Vitamin D

In our study sample, 36.5% (N = 81/222, 95% CI 30.1% to 43.2%) had vitamin D insufficiency/deficiency, with 63.1% having normal levels (Table 2). Looking at correlates for the presence of vitamin D insufficiency/deficiency, both older age and ethnicity were significant correlates (Table 3). An increase of 1 month in age was associated with an increase of 4% (95% CI 3% to 6%) in the odds of having abnormal vitamin D levels after adjusting for race, gender, and picky eating. As for ethnicity, children of Malay [OR = 5 (95% CI 1.6 to 15.9)], Indian [OR = 20.9 (95% CI 4.7 to 92.0)], and other ethnicities [OR = 3.2 (95% CI 1.01 to 10.4)] were all found to have higher odds of having abnormal vitamin D levels compared to those of Chinese ethnicity. It is noteworthy that the presence of picky eating was not associated with increased odds of vitamin D insufficiency/deficiency. However, amongst those with picky eating, it was found that those who were older had higher odds of vitamin D insufficiency (OR: 1.51, 95% CI 1.11 to 2.06, *p* = 0.009) after adjusting for race and gender.

### 3.3. Iron

Iron deficiency occurred in 37.7% of children (N = 89/236, 95% CI 31.5% to 44.2%), with iron deficiency anaemia found in 15.6% (N = 19/122). There were no significant correlates for iron deficiency identified (Table 4). Older children had higher odds of having iron deficiency anaemia (OR: 1.03, 95% CI 1.01 to 1.06; *p* = 0.016) after adjusting for race, gender, and picky eating (Table 5). However, amongst the picky eaters, there were no statistical association between age and iron deficiency anaemia (OR: 0.87, 95% CI 0.53 to 1.42, *p* = 0.577) after adjusting for race and gender. Of interest is the finding that the proportion of children who had iron deficiency amongst the picky eaters (44%, N = 38/87) compared with non-picky eaters (40%, N = 37/92) was similar.

## 4. Discussion

Our key study results indicate a high occurrence of both vitamin D and iron deficiency in a sizeable cohort of children with ASD in Singapore. More than a third of the sample had such deficiencies, with ethnic differences in the occurrence of vitamin D insufficiency/deficiency. It is also important to note that laboratory investigations for the presence of these deficiencies were only performed in approximately 23.8% (241/1014) of the children with ASD seen in the unit, so perhaps the true rates of such deficiencies could be different. To the best of our knowledge, this is the first descriptive study on nutritional deficiencies in children with ASD in Singapore.

The largest source of vitamin D is from cutaneous synthesis through exposure to direct sunlight. Singapore, being a tropical island, is not deprived of sunlight compared to temperate countries. Yet, the occurrence of vitamin D insufficiency/deficiency is high in our sample. While comparative data among non-autistic children in Singapore is not available, this is consistent with that from Singaporean adults and adults working indoors, which suggest deficiency rates of between 32.9% to 40% [26,27]. Our findings are, however, lower when compared to those from our neighbour Malaysia, where the rates were higher at 61% [11]. This could be due to varying ethnicity of the samples, with a predominance of Malay ethnicity in the Malaysian sample, which may be associated with cultural practices such as use of clothing that covers most part of the body which is common in this ethnicity. Regardless, vitamin D insufficiency/deficiency is common even in tropical Singapore.

Our data suggested significant ethnic differences in the presence of vitamin D insufficiency/deficiency, with Chinese ethnicity being a protective factor. This is likely related to the lighter pigmentation of skin in those of Chinese ethnicity, affecting the absorption of ultraviolet rays from the sun. This is in keeping with studies from Western countries, where the prevalence of Vitamin D deficiency was found to be higher in dark-skinned ethnic subgroups [5,28]. We also found that older children were at increased risk of vitamin D insufficiency/deficiency. This is similar to another study from Turkey [29] and could be related to reduced outdoor time in older children due to autism-related behavioural challenges, which become more impactful with age. This could also be related to commencement of formal schooling, leading to greater time spent within the classroom and less time outdoors. Among picky eaters, older children were also at increased risk of vitamin D insufficiency/deficiency. This may be due to caregivers supporting the diet of younger children with caloric fortification of foods and additional iron/vitamin D fortified formula milk. With age and decreasing dependence on formula milk, it becomes more challenging to provide a balanced diet, especially in children with selective eating. It is reported that low intake, genetic factors, and reduced sun exposure due to difficulties with social interaction and isolation contribute to this [4]. Physicians should consider these factors in their risk profiling of patients, and consider recommending children with ASD to undergo laboratory investigations for vitamin D deficiency, given our results and other studies suggesting higher prevalence of vitamin D deficiency in these individuals [30,31].

The iron deficiency rate in our study population was 37.7%, with 15.6% having iron deficiency anaemia. This is higher than the rates found in other countries such as the United States and Sydney [21,22]. Diet is the main source of iron and in autism, it was believed that selective eating (rejection of red meat due to its appearance and texture) [4] would increase the risk of iron deficiency. However, we found that picky eating was not a predictive factor for this. This could be the result of a difference in diet, as a large proportion of the Asian diet is made up of carbohydrates compared to among Caucasian populations, where red meats are more popular.

Age was not a significant predicator for iron deficiency alone which is similar to other reports, such as that of Dosman et al. (2006), where iron levels in preschool and school-aged autistic children were not different [32]. Amongst the picky eaters, there was also no association between age and iron deficiency. These findings could be related to the common use of formula milk when younger, which is fortified with nutrients including iron, thereby protecting against nutritional deficiencies.

In this study, picky eaters were determined by caregiver reports of having concerns of picky eating and further details regarding this (e.g., specific food group/variety intake) and objective determination of the presence or severity of selective feeding was not conducted [33]. This may explain why there were no differences seen in iron deficiency rates between the picky eating and the non-picky eating groups, as it was those with severe food selectivity that would have nutritional deficiencies in iron and vitamin D [34,35]. However, our results are similar to another local study that included only children with selective feeding, who had rates of 33.9% and 37.7% for iron and vitamin D insufficiency/deficiency, respectively [36]. This suggests that there could either be an under-reporting of selective eating in our sample or that the parents who agreed for blood tests had underlying feeding concerns that were not reported.

### Clinical Implications

Despite the study population being limited to a specific sample of children from a single unit, our results show that a significant proportion of almost 40% of children diagnosed with ASD who had blood investigations performed had vitamin D insufficiency/deficiency and/or iron deficiency. Though the prevalence of deficiencies cannot be inferred from this study, any deficiency detected is significant, as supplementation can possibly improve the overall health of these patients. As such, clinicians should consider screening for these deficiencies, as it would benefit these children if timely supplementations were given. Clinicians should include counselling regarding the role of these investigations as part of routine reviews. In addition, as blood draws may be extra challenging in children with ASD, we suggest incorporating visual schedules and social stories to make the process less traumatic; this would also encourage parental consent for investigations. Noting the disparity between those who had iron-level tests, and a complete blood count performed, it is important that both these tests are performed together to allow full interpretation of iron status. Future studies should include studying the relationship between these deficiencies and core autism symptoms and the effect of correcting these deficiencies with varying doses of supplementation.

However, this study is not without limitations. Firstly, our study sample is likely subject to sampling bias as it was based on children whose caregivers agreed for blood investigations to be performed. These parents could have been more concerned regarding their child’s dietary habits, and this could have motivated them to proceed with blood investigations. This sampling bias may have resulted in an overestimation of the rates of nutritional deficiency in this sample. Secondly, we did not have a comparison control group of children without ASD and hence were unable to make inferences about the comparative occurrence of these nutritional deficiencies between the two groups. Nonetheless, this descriptive study provides a starting point for further comparative studies to determine the true prevalence of nutritional deficiency in the children with ASD. Thirdly, as this is a retrospective review, analysis could only be performed on information that was available in medical records. This limited the ability to assess for all possible correlates, including detailed information on diet, frequency, and duration of outdoor time, as well as the extent of autism features and cognitive ability. Lastly, we were not able to establish the cause-and-effect relationship in terms of these deficiencies, as this was a cross-sectional study.

## 5. Conclusions

Though conclusions regarding the actual prevalence of these deficiencies cannot be determined through this study, vitamin D and iron deficiencies were commonly detected in this study population of children with ASD in Singapore. The presence of picky eating was not a reliable clinical marker for the presence of nutritional deficiencies. In clinical practice, we suggest that clinicians consider testing the vitamin D and iron levels in children with ASD, especially vitamin D in those who are older and of Indian ethnicity. Identifying the presence of such deficiencies and pursuing appropriate treatment for these can result in improvements in the overall health and development in these children.

## Figures and Tables

**Table 1:** Demographic characteristics.

	Total N = 241
Age, years	
Mean (sd)	4.21 (2.25)
Median (25th Percentile–75th Percentile)	4.00 (2.38 to 6.00)
Gender, *n* (%)	
Male	191 (79.3%)
Female	50 (20.7%)
Race, *n* (%)	N = 191
Chinese	123 (64.4%)
Malay	23 (12.0%)
Indian	20 (10.5%)
Others	25 (13.1%)

**Table 2:** Occurrence of vitamin D and iron deficiency.

Category	N (%)
**Vitamin D (Total N = 222)**	
Deficient	10 (4.5%)
Insufficient	71 (32%)
Sufficient	140 (63.1%)
Toxicity	1 (0.5)
**Iron (Total N = 236)**	
Iron deficiency	89/236 (37.7%)
Iron deficiency anaemia	19/122 (15.6%)

**Table 3:** Correlates of presence of vitamin D insufficiency or deficiency ^1^.

	Presence of Vitamin D		Unadjusted		Adjusted	
	Insufficiency/ Deficiency (n = 81)	Sufficient/ Toxic (n = 141)	OR (95% CI)	*p*-Value	OR (95% CI)	*p*-Value
**Age (months)**						
Mean (SD)	58.3 (27.9)	46.7 (24.8)	1.02 (1.01 to 1.03)	0.002	1.04 (1.03 to 1.06)	<0.001
**Race**						
Malay	9 (14.5%)	12 (10.2%)	2.33 (0.72 to 7.52)	0.255	5.0 (1.6 to 15.9)	0.007
Indian	15 (24.2%)	4 (3.4%)	11.6 (2.8 to 49.1)	<0.001	20.9 (4.7 to 92.0)	<0.001
Other Races	9 (14.5%)	12 (10.2%)	2.33 (0.72 to 7.52)	0.255	3.2 (1.01 to 10.4)	0.048
Chinese	29 (46.8%)	90 (76.3%)	1		1	
**Gender**						
Male	63 (77.8%)	113 (80.1%)	0.87 (0.45 to 1.69)	0.676	1.52 (0.55 to 4.25)	0.422
Female	18 (22.2%)	28 (19.9%)	1		1	
**Eating Habit**						
Picky Eater	26 (44.8%)	54 (48.6%)	0.86 (0.45 to 1.62)	0.637	0.48 (0.21 to 1.08)	0.075
Non-Picky Eater	32 (55.2%)	57 (51.4%)	1		1	

^1^ reference being Chinese ethnicity.

**Table 4:** Correlates of presence of iron deficiency.

	Iron Deficiency		Unadjusted		Adjusted	
	Yes (n = 89)	No (n = 147)	OR (95% CI)	*p*-Value	OR (95% CI)	*p*-Value
**Age** (months)				0.809		0.212
Median (Min–Max)	54 (8–114)	44 (6–126)	1.00 (0.99 to 1.01)		0.99 (0.98 to 1.01)	
**Race**						
Malay	12 (15.2%)	11 (10.2%)	1.91 (0.64 to 5.72)	0.474	1.87 (0.73 to 4.78)	0.194
Indian	10 (12.7%)	9 (8.3%)	1.94 (0.59 to 6.39)	0.543	1.59 (0.52 to 4.90)	0.420
Other Races	13 (16.5%)	11 (10.2%)	2.07 (0.70 to 6.09)	0.321	1.95 (0.76 to 5.03)	0.168
Chinese	44 (55.7%)	77 (71.3%)	1		1	
**Gender**						
Male	69 (77.5%)	119 (81.0%)	0.81 (0.43 to 1.55)		0.94 (0.42 to 2.10)	
Female	20 (22.5%)	28 (19.0%)	1		1	
**Eating Habit**						
Picky Eater	38 (50.7%)	49 (47.1%)	1.15 (0.64 to 2.09)		1.17 (0.62 to 2.23)	
Non-Picky Eater	37 (49.3%)	55 (52.9%)	1		1	

**Table 5:** Correlates of presence of iron deficiency anaemia.

	Iron Deficiency Anaemia		Unadjusted		Adjusted	
	Yes (n = 19)	No (n = 103)	OR (95% CI)	*p*-Value	OR (95% CI)	*p*-Value
**Age (months)**						
Median (Min–Max)	70 (18–95)	44 (12–114)	1.03 (1.01 to 1.05)		1.03 (1.01 to 1.06)	
**Race**						
Malay	1 (5.3%)	17 (16.5%)	0.47 (0.03 to 6.47)	1	0.40 (0.04 to 3.66)	0.418
Indian	4 (21.1%)	10 (9.7%)	3.20 (0.60 to 17.11)	0.290	3.16 (0.69 to 14.45)	0.139
Other Races	6 (31.6%)	12 (11.7%)	4 (0.90 to 17.85)	0.080	2.02 (0.48 to 8.42)	0.337
Chinese	8 (42.1%)	64 (62.1%)	1		1	
**Gender**						
Male	13 (68.4%)	86 (83.5%)	0.43 (0.14 to 1.29)	0.130	0.47 (0.13 to 1.64)	0.235
Female	6 (31.6%)	17 (16.5%)	1		1	
**Eating Habit**						
Picky Eater	9 (50.0%)	41 (45.1%)	1.22 (0.44 to 3.36)	0.701	0.67 (0.20 to 2.30)	0.528
Non-Picky Eater	9 (50.0%)	50 (54.9%)	1		1	

## Data Availability

The data is available upon reasonable request.

## Supplementary Materials

The following supporting information can be downloaded at: https://www.mdpi.com/article/10.3390/nu17172738/s1, Table S1: Demographic characteristics of children with and without blood investigations.

## Abbreviations

The following abbreviations are used in this manuscript:

ADOS-2	Autism Diagnostic Observation Schedule
[25(OH)D]	25-hydroxyvitamin D
