# Supplementary material for: Occurrence and Correlates of Vitamin D and Iron Deficiency in Children with Autism Spectrum Disorder
Source: Nutrients. 2025 Aug 23;17(17):2738. doi: 10.3390/nu17172738 (PMC12429854; doi:10.3390/nu17172738)
Supplement: Supplementary file 1 [file nutrients-17-02738-s001.zip › nutrients-3800184-supplementary.pdf]

Supplementary Table S1. Demographic characteristics of children with and without blood investigations

	Either Vit D or Iron Investigation		p-value
	Done<br>N = 241	Not Done<br>N = 773	
Age, years			< 0.001
Mean (sd)	4.21 (2.25)	4.94 (1.91)	
Gender, n (%)			0.783
Male	191 (79.3%)	619 (80.1%)	
Female	50 (20.7%)	154 (19.9%)	
Race, n (%)	191	773	
Chinese	123 (64.4%)	467 (60.4%)	0.145
Malay	23 (12.0%)	141 (18.2%)	
Indian	20 (10.5%)	60 (7.8%)	
Others	25 (13.1%)	105 (13.6%)	
Picky Eater	182	497	
Yes	89 (48.9%)	138 (27.8%)	< 0.001
No	93 (51.1%)	359 (72.2%)	
